# Norepinephrine Stimulates Mobilization of Endothelial Progenitor Cells after Limb Ischemia

**DOI:** 10.1371/journal.pone.0101774

**Published:** 2014-07-09

**Authors:** Qijun Jiang, Shifang Ding, Jianxiang Wu, Xing Liu, Zonggui Wu

**Affiliations:** 1 Department of Cardiology, Wuhan General Hospital of Guangzhou Military, Wuhan, Hubei, China; 2 Department of Cardiology, Changzheng Hospital, Second Military Medical University, Shanghai, China; University of Frankfurt - University Hospital Frankfurt, Germany

## Abstract

**Objective:**

During several pathological processes such as cancer progression, thermal injury, wound healing and hindlimb ischemia, the mobilization of endothelial progenitor cells (EPCs) mobilization was enhanced with an increase of sympathetic nerve activity and norepinephrine (NE) secretion, yet the cellular and molecular mechanisms involved in the effects of NE on EPCs has less been investigated.

**Methods and Results:**

EPCs from BMs, peripheral circulation and spleens, the VEGF concentration in BM, skeletal muscle, peripheral circulation and spleen and angiogenesis in ischemic gastrocnemius were quantified in mice with hindlimbs ischemia. Systemic treatment of NE significantly increased EPCs number in BM, peripheral circulation and spleen, VEGF concentration in BM and skeletal muscle and angiogenesis in ischemic gastrocnemius in mice with hind limb ischemia, but did not affair VEGF concentration in peripheral circulation and spleen. EPCs isolated from healthy adults were cultured with NE in vitro to evaluate proliferation potential, migration capacity and phosphorylations of Akt and eNOS signal moleculars. Treatment of NE induced a significant increase in number of EPCs in the S-phase in a dose-dependent manner, as well as migrative activity of EPCs in vitro (p<0.05). The co-treatment of Phentolamine, I127, LY294002 and L-NAME with NE blocked the effects of NE on EPCs proliferation and migration. Treatment with NE significantly increased phosphorylation of Akt and eNOS of EPCs. Addition of phentolamine and I127 attenuated the activation of Akt/eNOS pathway, but metoprolol could not. Pretreatment of mice with either Phentolamine or I127 significantly attenuated the effects of NE on EPCs in vivo, VEGF concentration in BM, skeletal muscle and angiogenesis in ischemic gastrocnemius, but Metoprolol did not.

**Conclusion:**

These results unravel that sympathetic nervous system regulate EPCs mobilization and their pro-angiogenic capacity via α adrenoceptor, β 2 adrenoceptor and meanwhile Akt/eNOS signaling pathway.

## Introduction

Mobilization and recruitment of endothelial progenitor cells (EPCs) in response to disease or tissue injury, such as cancer progression[1], thermal injury[2], wound healing[3] and hindlimb ischemia[4], are of paramount importance and occupy a predominant hierarchical role in the orchestration of tissue remodeling after ischemia. The therapeutic application of EPCs is widely anticipated. Currently, however, there are both practical and technical complications associated with harvesting, isolation, ex vivo expansion, and delivery of EPCs. An alternative strategy for EPCs therapy is to stimulate the mobilization of EPCs from the bone marrow (BM) into the circulation, thereby circumventing these issues.

Maintenance and mobilization of progenitor cells in BM are controlled by various cytokinesincluding vascular endothelial growth factor (VEGF)[5], colony-stimulating factors[6] and angiogenic cytokines1[7]. During hindlimb ischemia, catecholamines have been proposed to contribute to collateral growth and angiogenesis in ischemic tissue. [8] It is now well established that BM and secondary lymphoid tissues are innervated by noradrenergic sympathetic nerve fibers, which release catecholamines from the sympathetic nerve terminals. Recent evidence suggests that catecholamines are also able to control BM derived cells mobilization.[9] Administration of a β2 adrenergic agonist enhances mobilization of progenitor cells in both control and norepinephrine (NE)-deficient mice. Moreover, inhibition of adrenergic neurotransmission reduces hematopoietic stem cell mobilization. In line with these results, adenoviral-mediated gene transfer of the human β2 adrenergic receptor to the endothelium of the rat femoral artery results in ameliorated angiographic blood flow and hindlimb perfusion after chronic ischemia, whereas angiogenesis is severely impaired in β2 adrenergic receptor-deficient mice subjected to femoral artery resection. However, whether NE could influence mobilization of BM-derived angiogenic EPCs had less been investigated. In this study, we have identified pathways that NE regulates the mobilization of EPCs in mice with hindlimb ischemia.

## Methods

### Hind limb ischaemia model

All procedures involving animals were performed in accordance with the recommendations in the Guide for the Care and Use of Laboratory Animals of Second Military Medical University and approved by the Committee on the Ethics of Animal Experiments of Changzheng Hospital. All surgery was performed under anesthesia by intraperitoneal injection with a mixture of ketamine (80 mg/kg) and xylazine (4 mg/kg), and all efforts were made to minimize suffering. Experiments were performed in Male C57BL/6J mice (7–8 weeks old, 15–19 g, Shanghai Experimental Animal Center of the Chinese Academy of Sciences, Shanghai, China). Hind limb ischemia was induced by unilateral resection of the left femoral artery from the proximal end of the femoral artery up to the distal portion of the saphenous vein. The femoral artery and all side-branches were dissected and excised, then the overlying skin was closed using a surgical stapler. Mice were sacrificed with an overdose of the same anesthetic.Bblood was withdrawn for the FACS analysis (EDTA-anti-coagulated) by the heart puncture and the spleen and the tibia and femur of both legs were respectively kept for isolation of splenic cells and BM cells.

### Flow cytometric analysis

To quantify EPCs number, cells from peripheral blood, BM homogenates and splenic tissue homogenates were lysed and used for flow cytometric analysis. All procedures were performed according to the manufacturer's instructions. After 30 min incubation with FITC-conjugated anti-mouse CD34 (BD Biosciences), PE-conjugated anti-mouse Flk-1 (BD Biosciences) and APC-conjugated anti-mouse CD45 (BD Biosciences), cells were washed with PBS and fixed in 4% paraformaldehyde and analyzed by Flow Cytometry (Miltenyi Biotec, Bergisch Gladbach, Germany). Staining was performed in the presence of saturating concentrations of rat monoclonal unconjugated antibodies against Fc receptors (anti-CD16/32, BD Bioscience) to reduce nonspecific binding. Isotype-identical antibodies served as controls (IgG1-PE and IgG2a-FITC, BD Bioscience). Each analysis included 100,000 events. Data were analyzed using MACSQuantify Software (Miltenyi Biotec). The number of CD34/Flk-1 double positive and CD45 negative cells was counted as EPCs.

### Measurement of capillary density in the ischemic limb

Capillary densities of ischemic skeletal muscle tissues were analyzed at the level of the microcirculation. At day 5 after the induction of ischemia, mice were sacrificed with overdose of anesthetic and the gastrocnemius muscles were harvested, embedded in OCT compound (Leica) and snap frozen in liquid nitrogen. Frozen 10 µm-thick sections of the distal part of the gastrocnemius muscle were fixed in ice-cold acetone for 10 min, incubated for 1 h with a rat anti-mouse CD31 monoclonal antibody (Abcam), and then incubated with a goat anti-rat secondary antibody coupled to FITC (Abcam). Sections were examined by a blinded observer using a Fluorescence microscopy (Olympus). Capillaries were identified by positive staining for CD31. Ten different fields from each tissue preparation were randomly selected, and visible capillaries were counted. Capillary density was expressed as the number of capillaries per muscle bundle.

### Quantification of VEGF

The blood serum, bone marrow, spleen and ischemic muscle were used to measure VEGF concentration. The BM homogenates, splenic homogenates and skeletal muscle homogenates were suspended at 1 ml of 10 mM Tris–HCl (pH 7.4) and centrifuged at 15 000× g at 4°C for 15 min. The supernatant was passed through a Microcon YM-10 filter (Millipore) at 5 000 × g at 4°C for 60 min. VEGF concentration was determined in supernatants by specific enzyme-linked immunosorbent assays (ELISAs) (Biorbyt).

### Preparation of human EPCs

EPCs were prepared as previously described. [10] Peripheral Blood Mononuclear Cells (PBMCs) were isolated from peripheral blood of healthy human volunteers as described by Sun et al by density-gradient centrifugation with Ficoll. Immediately after isolation, total MNCs (5*106 cells/mL medium) were plated on culture dishes coated with human fibronectin (Sigma) and maintained in endothelial basal medium (EBM, CellSystems) supplemented with EGM SingleQuots, VEGF (10 ng/mL) and 20% fetal bovine serum. The medium was replaced every 3 days. Adherent cells on 8 days of culture were stained by acetylated LDL and were labeled with DiI (DiI-acLDL, Biomedical Technologies) and fluorescein isothiocyanate (FITC)-labeled lectin from ulex europaeus (Sigma). Double-positive cells for DiI-acLDL and FITC-labeled lectin were identified as EPCs, as reported previously. All human researches were conducted in Changzheng Hospital. All procedure were permitted by the human volunteers with written consent and approved by the Committee on the Ethics of human Experiments of Changzheng Hospital.

### EPCs proliferation assay

EPCs (1,000 cells/well), seeded on 96-well plates (experiments were performed in six copies), were treated with EBM-2 (200 µl/well) or drugs, such as NE, Phentolamine, Metoprolol, or I127 for various interventions. After incubation for 20 h, 10 µM 5-bromo-2′-deoxyuridine (BrdU) labeling solution (from a Cell Proliferation ELISA kit, Roche Applied Science) was added into each well, and the incubation was continued for another 4 h. Cells were fixed and exposed to anti-BrdU and substrate solutions. The reaction product was quantified by measuring the absorbance using a multiwell spectrophotometer (Spectromax340 PC, Molecular Devices).

### EPC migration assay

The migratory function of EPCs was evaluated with transwell chamber assay as described before[11]. In brief, EPCs were detached with trypsin and EPCs (3×104) in 100 µl EGM-2 media (0.5% FBS) were placed in 24 well transwell upper chamber (8 m pore size). Then, 600 µl EGM-2 media containing 0.5% FBS were placed into the lower chamber. After 24 h incubation at 37°C, EPCs on the top of the membrane were wiped off with a cotton swab. The membrane of transwell filter was stained by with 1% crystal violet solution and washed with PBS. Migration of EPCs was evaluated by measuring the migrated cells in six random high-power (100×) microscope fields, and the average of these six fields was taken. Experiments were repeated six times.

### Western blot analysis

After 7 days of culture, EPCs were deprived from serum for 12 h, and then challenged with drugs, such as NE, Phentolamine, Metoprolol, or I127 for various interventions. Cells were lysed in protein extract buffer (1 ml protein extract buffer with 5 ml mixture of protease inhibitors, 5 ml PMSF and 5 ml mixture of phosphatases) at 4°C with sonication for 30 min. The lysates were centrifuged at 14 000×g and 4°C for 30 min. Loading buffer was added to each volume and boiled for 10 min. Samples were resolved on 12% SDS-PAGE and electro-transferred onto a nitro-cellulose membrane. The membrane was blocked with 5% non-fat milk. Blots were incubated with mouse anti-eNOS IgG, mouse anti- phosphorylated eNOS IgG, mouse anti- Akt IgG and mouse anti- phosphorylated Akt IgG each at a dilution of 1∶1000 for 12 h at 4°C. The blots were washed in TBS/T (Tris-buffered saline containing 0.2% Tween 20) and exposed to HRP-conjugated anti-goat IgG or anti-mouse secondary antibody (1∶5000) for 1 h, respectively, and then visualized by enhanced chemiluminescence detection reagents. The signal intensity of blotting was normalized to the Western signal of the corresponding total protein. Relative intensities of protein bands were analyzed by Image-pro plus6.0 (Media Cybernetics, Silver Spring, MD, USA).

### Statistical analysis

All data were expressed as mean ± SEM. Statistical analysis between two groups was performed using unpaired Student's t test and comparisons between multiple groups were made by one-way ANOVA. Probability values were considered significant at p<0.05.

## Results

### NE increased mobilization of EPCs in limb ischemia mouse

**Figure 1:**
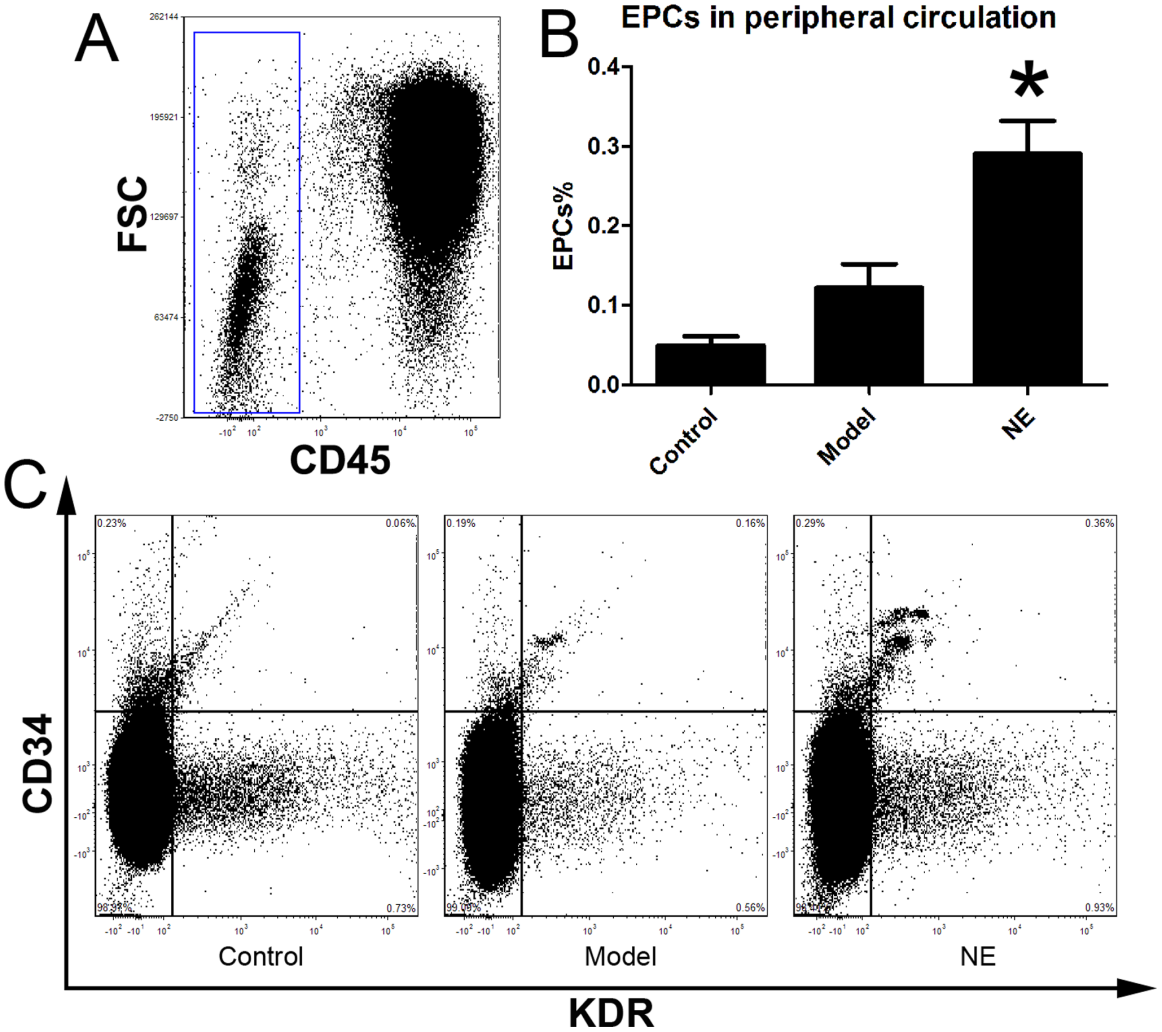
NE increased EPCs in peripheral blood in mice with limb ischemia. Cells from peripheral blood were lysed and analyzed by flow cytometry. Cells were sequentially gated based on CD45 (A), CD34 and KDR expression (C). Circulating EPCs were defined as CD45-/CD34+/KDR+ cells. A gate was used to select the total CD45- cell population (A). Corresponding flow cytometric analysis was used to detect CD34+/KDR+ cells in the gated CD45- cell population. Proportion of EPCs in peripheral blood was increased from 0.15±0.04% to 0.31±0.05% after intraperitoneal injection of NE in limb ischemia model (B, * P<0.05 compared with the model group). Representative flow cytometric analysis of EPCs (CD34/Flk-1 cells) were showed in part C.

**Figure 2:**
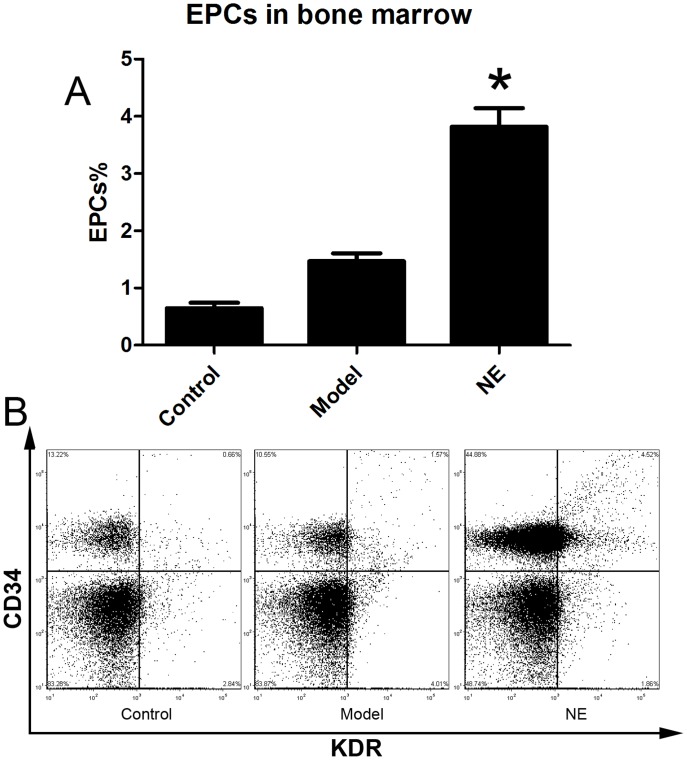
NE increased EPCs in BM in mice with limb ischemia. Cells from peripheral blood were lysed and analyzed with flow cytometry. BM derived EPCs were defined as CD34+/KDR+ cells (B). Proportion of EPCs in BM was increased from 1.47±0.29% to 4.23±1.01% after intraperitoneal injection of NE in mice with limb ischemia (A, * P<0.05 compared with model group). Representative flow cytometric analysis of EPCs (CD34+/Flk-1+cells) were showed in part B.

**Figure 3:**
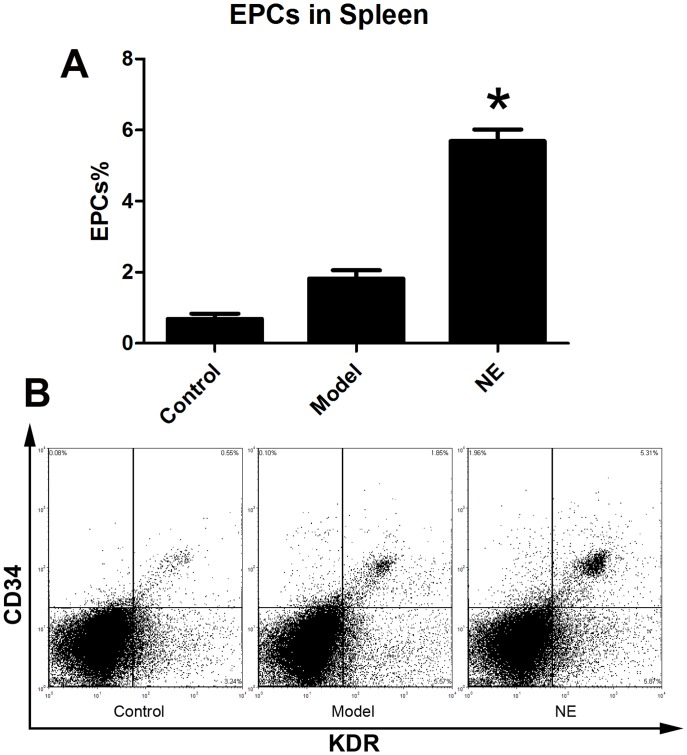
NE increased EPCs in spleen in mice bearing limb ischemia. Cells from splenic tissue homogenates were lysed and analyzed with flow cytometry. EPCs in spleen were defined as CD34+/KDR+ cells (B). Proportion of EPCs in spleen was increased from 1.94±0.39% to 4.89±0.36% after intraperitoneal injection of NE in mice with limb ischemia (A, * P<0.05 compared with model group). Representative flow cytometric analysis of EPCs (CD34+/Flk-1+cells) were showed in part B.

Male C57BL/6J mice were randomized in three groups. Limb ischemia model was (n = 6) prepared in model group and NE treatment group as describe before. NE 1 mg (kg body weight)-1 day-1 was firstly intraperitoneally injected in NE treatment group 24 h after the resection of the left femoral artery, and each 24 h later. At the 5th day, cells from peripheral blood, BM homogenates and splenic tissue homogenates were lysed and used for flow cytometric analysis. The number of CD34/Flk-1 positive and CD45 negative cells in the peripheral circulation were quantified with FCS analyzer. The proportion of EPCs in peripheral blood was increased from 0.15±0.04% to 0.31±0.05% in NE treated group compared to limb ischemia group (Figure 1). Likewise, the number of CD34/Flk-1 positive cells in BM and spleen was detected. The proportion of EPCs in BM and spleen respectively was increased from 1.47±0.29% to 4.23±1.01% and from 1.94±0.39% to 4.89±0.36% in NE group compared to model group (Figure 2, Figure 3).

### NE increased Capillary density in the ischemic gastrocnemius

**Figure 4:**
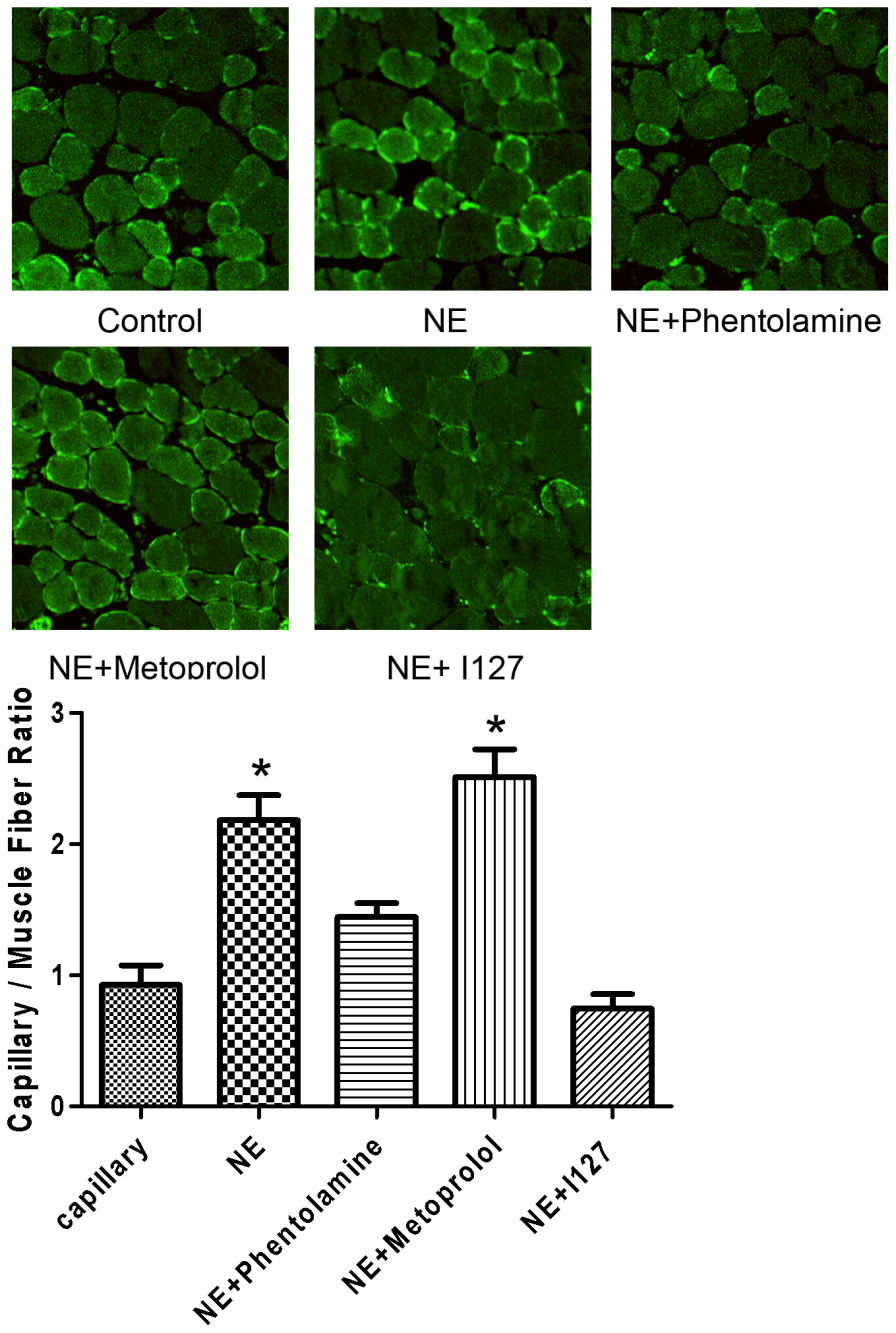
The angiogenesis in the caudal gastrocnemius was augmented by NE and blocked by Phentolamine and 127 in vivo. Cells positive for CD31 and muscle fiber number were both counted. The ratio of capillary number to muscle fiber number was determined. NE significantly increased hind-limb vascularization as assessed by capillary densitometry (2.35-fold increase relative to PBS-treated mice; P<0.01, Figure 4). The increase of neovessel formation in ischaemic muscles was inhibited by co-treatment of Phentolamine (1.56-fold increase relative to PBS-treated mice; P<0.01) and selective β 2 adrenoceptor blocker I127 (0.81-fold increase relative to PBS-treated mice; P<0.01), but not Metoprolol (2.71-fold increase relative to PBS-treated mice; P<0.01).

To determine if NE stimulates neovascularization in the setting of ischemia, we examined the angiogenesis in the caudal gastrocnemius. Muscle atrophy from ischemia and/or reduced use can confound the interpretation of changes in capillary density, as opposed to angiogenesis. Therefore, the ratio of capillary number to muscle fiber number was determined. Histological analysis of the ischemic tissues with CD31, an endothelial cell marker, demonstrated that NE significantly increased hind-limb vascularization (2.35-fold increase relative to PBS-treated mice; P<0.01, Figure 4). The increase of neovessel formation in ischaemic muscles was inhibited by co-treatment of Phentolamine (1.56-fold increase relative to PBS-treated mice; P<0.01) and selective β 2 adrenoceptor blocker I127 (0.81-fold increase relative to PBS-treated mice; P<0.01), but not Metoprolol (2.71-fold increase relative to PBS-treated mice; P<0.01). These results suggested that activation of α adrenoceptor and β 2 adrenoceptor results in increases of angiogenesis of ischemic limb.

### NE increased production of vascular endothelial growth factor

**Figure 5:**
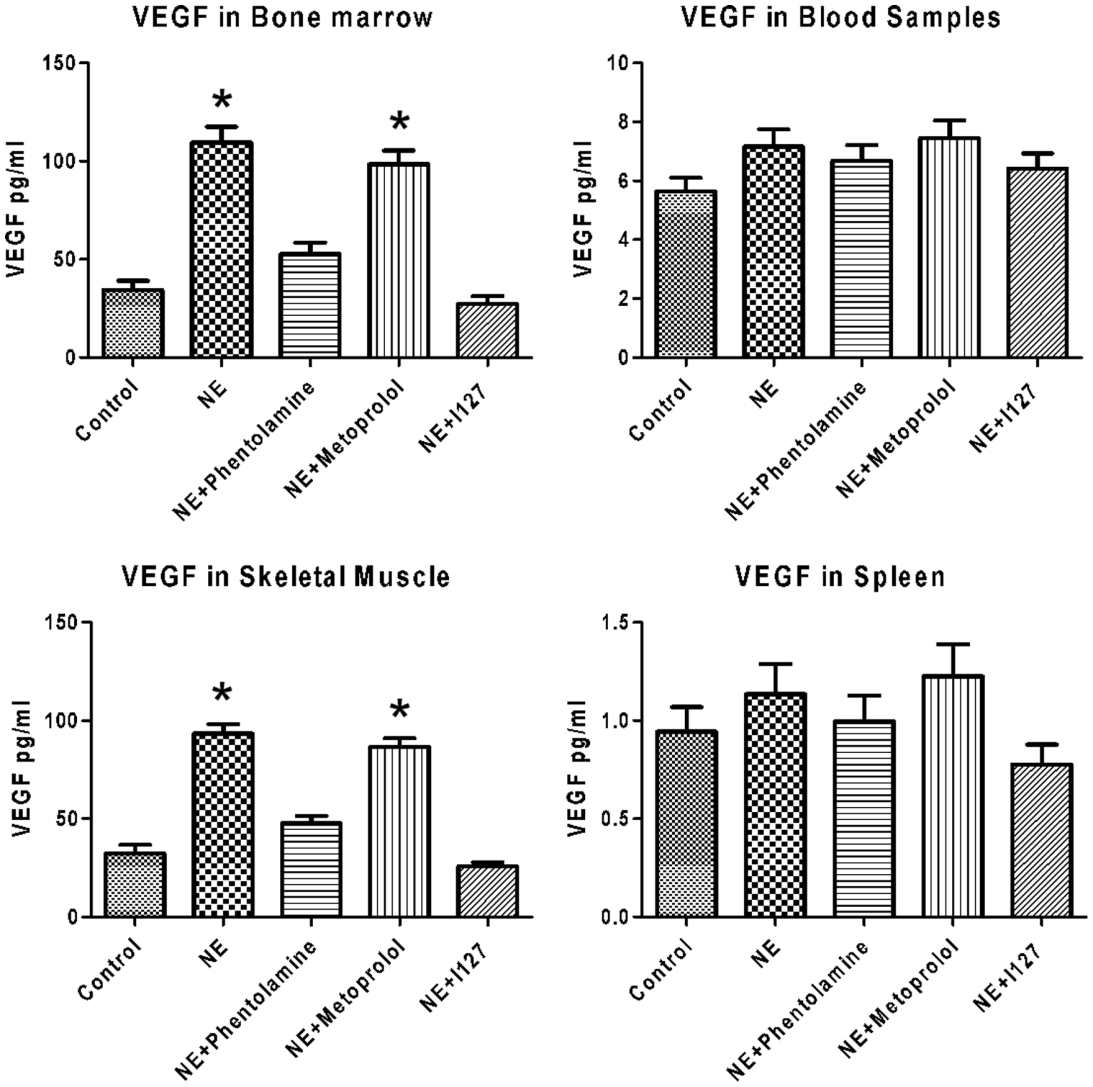
Effects of NE on VEGF production. The blood samples, BM homogenates, skeletal muscle and splenic tissue homogenates were collected from limb ischemic mice and measured by ELISA. NE significantly increased VEGF concentrations in both skeletal muscle and blood sumples, but did not affect it in bone marrow and spleen. Co-treatment of Phentolamine, I127attenuated the increase of VEGF in bone marrow and skeletal muscle, but did not affect it in blood samples and spleen.

VEGF have been suggested as mediators of EPC regulation. The blood samples, BM homogenates, skeletal muscle homogenates and splenic tissue homogenates were collected from limb ischemic mice and processed for VEGF analysis. Results showed that NE treatment increased VEGF concentrations in both bone marrow and skeletal muscle, but did not affect it in blood sumples and spleen. Co-treatment of Phentolamine, I127attenuated the increase of VEGF in bone marrow and skeletal muscle, but did not affect it in blood samples and spleen (Figure 5).

### Norepinephrine stimulates proliferation and migration of EPCs in vitro

**Figure 6:**
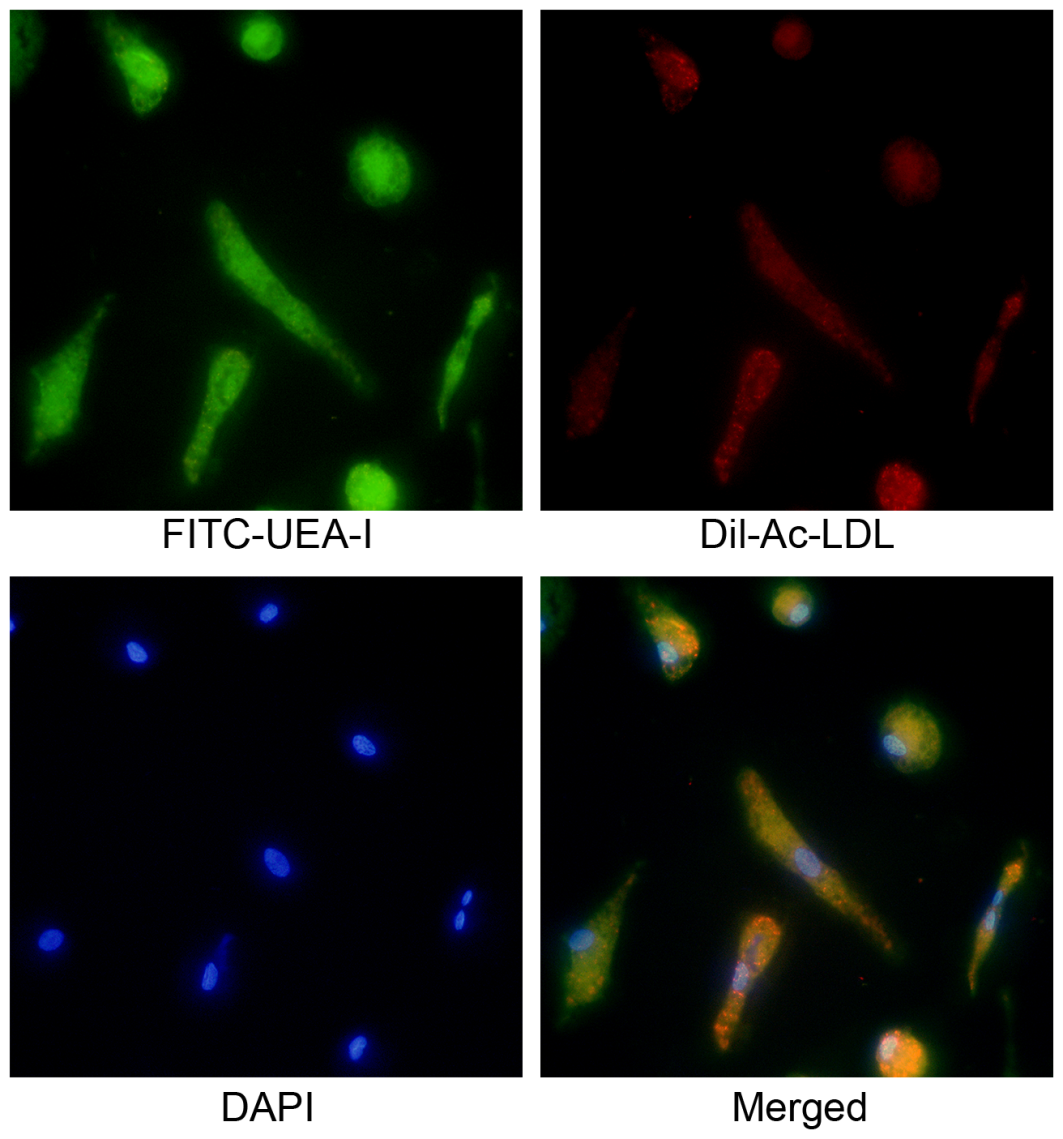
Characterization of EPCs derived from human peripheral circulation. EPCs exhibitedspindle-shaped or cobblestone-like morphology and were stained by DAPI and double labeled by Dil-Ac-LDL and FITC-UEA-I.

**Figure 7:**
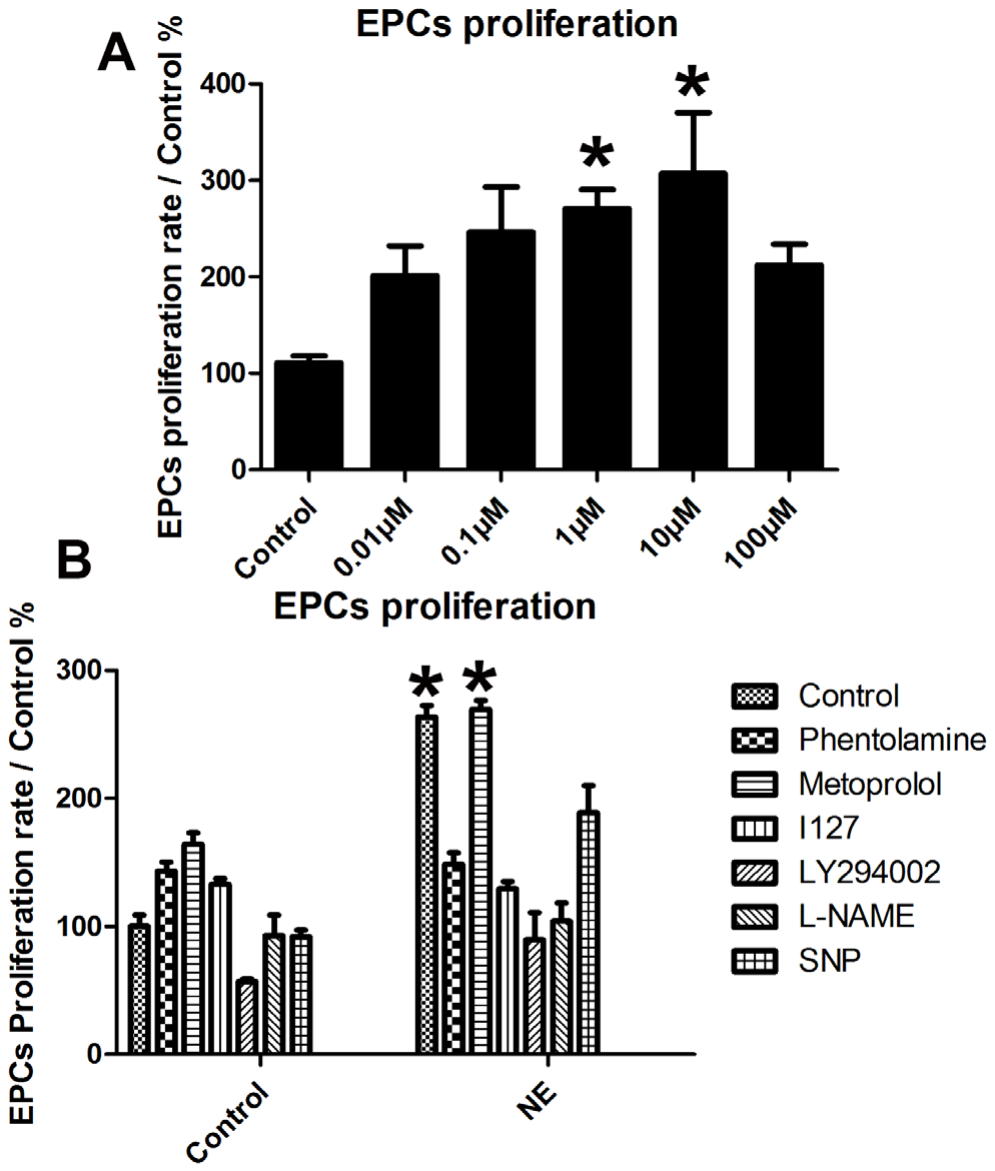
Proliferation potential of EPCs was increased by NE and blocked by Phentolamine, I127, LY294002 and L-NAME in vitro. Growth capacity of PBMC-derived EPCs after 8 days of culture was evaluated (n = 6). As shown by multiwell spectrophotometer, the treatment of NE induced a significant increase of the number of EPCs in the S-phase in a dose-dependent manner, as well as proliferation of EPC (* P<0.05 compared with model group). Pretreatment of phentolamine, I127, LY294002 and L-NAME significantly blocked the effect of NE on EPCs proliferation (Figure 5), but Metoprolol could not.

**Figure 8:**
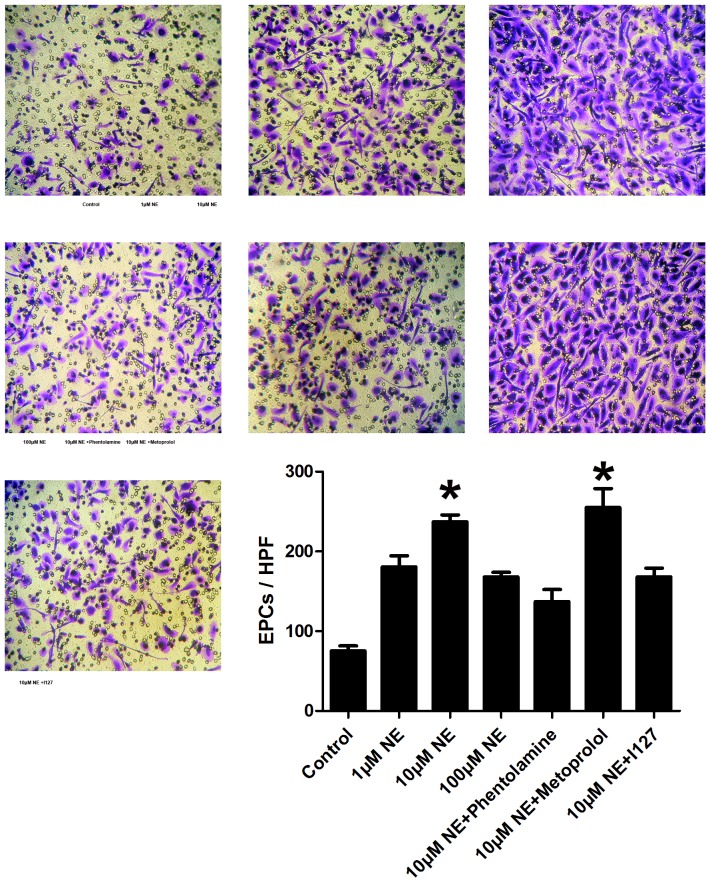
Migrative activity of EPCs was increased by NE and blocked by Phentolamine and 127 in vitro. The migrative activity of EPCs was detected using transwell chamber assay. EPCs stayed on the membrane in the upper chamber were wiped off with a cotton swab. EPCs stayed on the lower membrane of transwell filter were stained with 1% crystal violet solution and counted in six random high-power (100×) microscope fields. NE increased migration of EPCs in a dose dependent manner (* P<0.05 compared with control group). This effect could be blocked by either pretreatment of phentolamine or I127, but not Metoprolol. Representative figure of EPCs passed through the holes into the lower chamber of each group are showed here.

The EPCs derived from PBMCs of normal healthy donor were used to assay the proliferation potential and migration of EPCs. After 8-day culture of mononuclear cells, spindle-shaped or cobblestone-like adherent cells were observed. Most of the adherent cells were double stained by DiI-acLDL and FITC-labeled lectin (Figure 6). As shown by multiwell spectrophotometer of BrdU-labeled cells, the treatment of NE induced a significant growth in number of EPCs in the S-phase in a dose-dependent manner, as well as proliferation of EPCs (Figure 7, p<0.05). The migrative activity of EPCs was detected using transwell chamber assay. The results demonstrated that NE significantly increased migration of EPCs in a dose dependent manor (Figure 8, p<0.05).

### Norepinephrine increased proliferation and migration of EPCs via α adrenoceptor, β 2 adrenoceptor and Akt/eNOS signaling pathway in vitro

To evaluate the possible molecule mechanism, adrenoceptor blockers (Phentolamine, Metoprolol and selective β 2 adrenoceptor blocker I127), PI3K inhibitor LY294002, eNOS inhibitor L-NAME and NO donor SNP were pretreated to EPCs 1 h before the addition of NE to culture media. Phentolamine, I127, LY294002 and L-NAME significantly blocked effect of NE on EPCs proliferation (Figure 7). Similar results were showed by transwell chamber assay. Pretreatment of Phentolamine and I127 decreased EPCs migrative activity (Figure 8). These results suggested that NE increased EPCs mobilization probably via α adrenoceptor, β 2 adrenoceptor and Akt/eNOS signaling pathway.

### Inhibition of α adrenoceptor and β 2 adrenoceptor attenuated the elevation of EPCs in vivo

**Figure 9:**
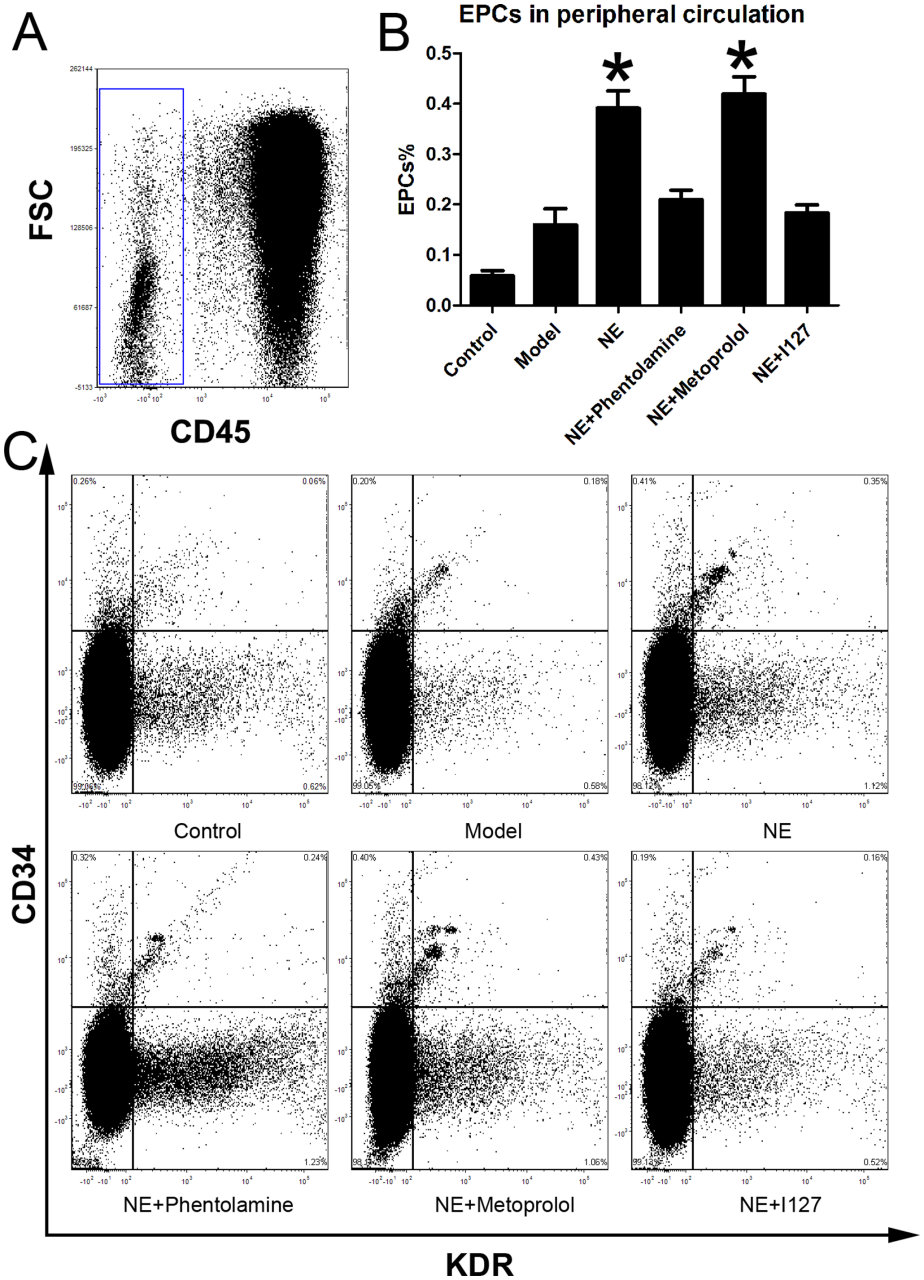
Inhibition of α adrenoceptor and β 2 adrenoceptor attenuated the elevation of the proportion of EPCs in peripheral circulation. Cells from peripheral blood were lysed and analyzed with flow cytometry. Cells were sequentially gated based on CD45 (A), CD34 and KDR expression (C). Circulating EPCs were defined as CD45-/CD34+/KDR+ cells. A gate was used to select the total CD45- cell population (A). Corresponding flow cytometric analysis was used to detect CD34+/KDR+ cells in the gated CD45- cell population. Co-treatment of either phentolamine or I127 with NE significantly attenuated the increases of EPCs in peripheral circulation, but Metoprolol didn't (B, *P<0.05 compared with the model group.). Representative flow cytometric analysis of EPCs (CD34/Flk-1 cells) were showed in part C.

**Figure 10:**
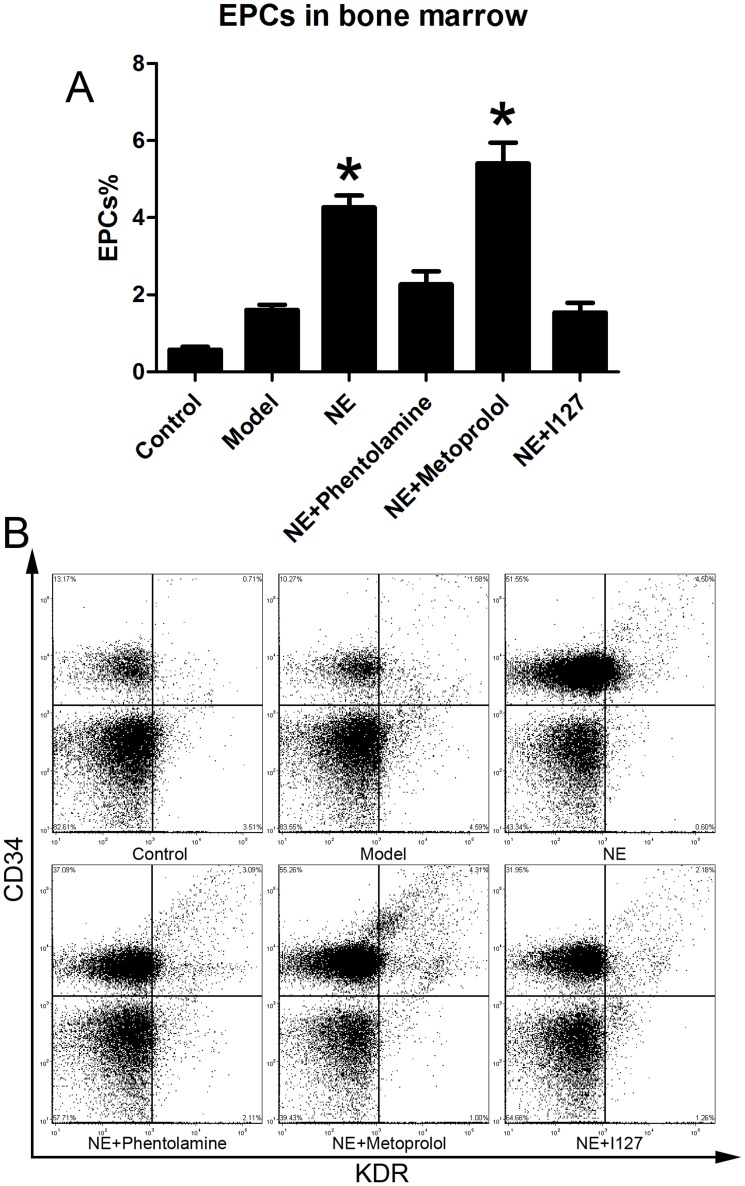
Inhibition of α adrenoceptor and β 2 adrenoceptor attenuated the elevation of EPCs in BM. Cells from BM homogenates were lysed and analyzed with flow cytometry. BM derived EPCs were defined as CD34+/KDR+ cells (B). Co-treatment of either Phentolamine or I127 with NE significantly attenuated the increases of EPCs in peripheral circulation, but Metoprolol didn't (B, *P<0.05 compared with model group). Representative flow cytometric analysis of EPCs (CD34+/Flk-1+cells) were showed in part B.

**Figure 11:**
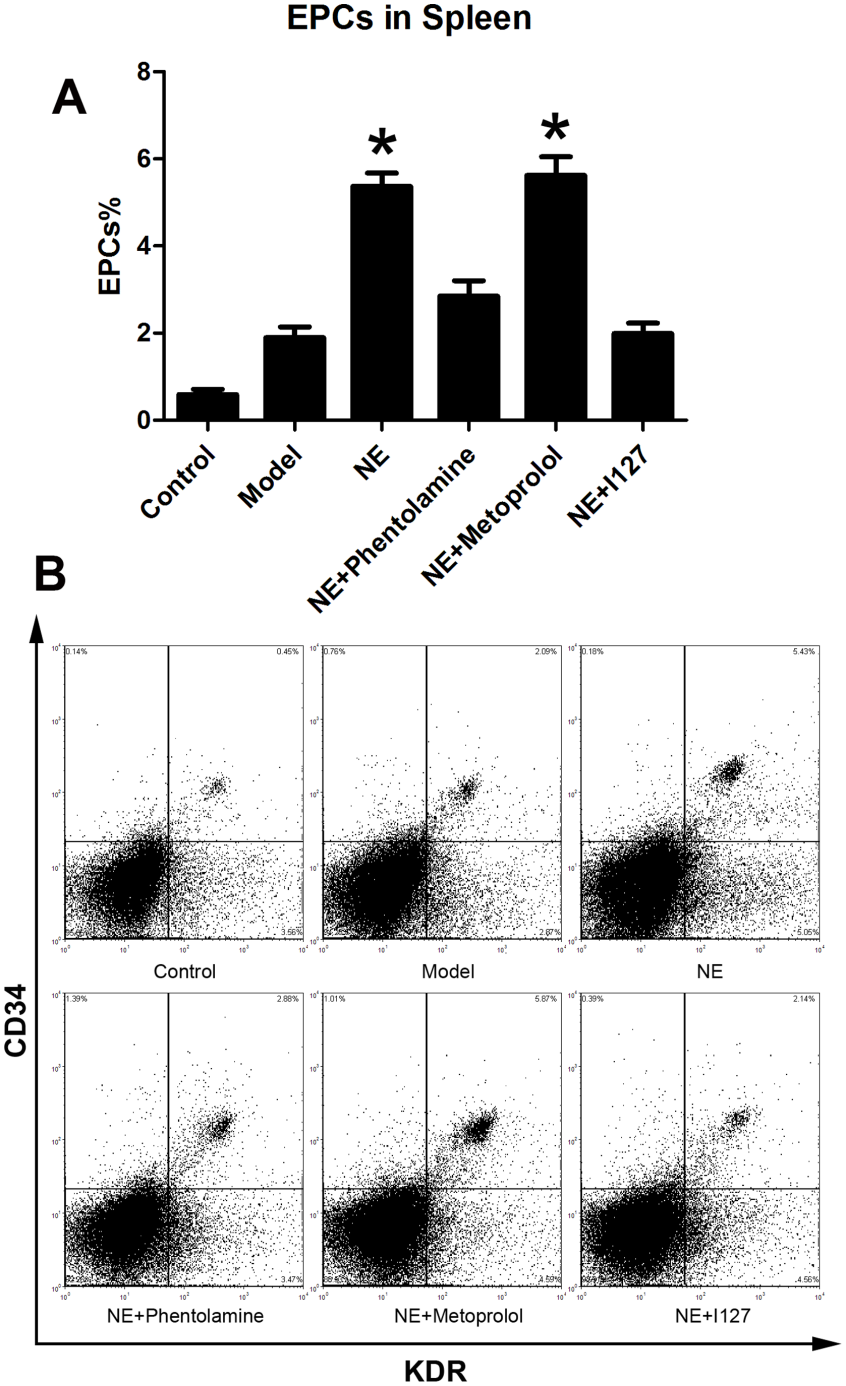
Inhibition of α adrenoceptor and β 2 adrenoceptor attenuated the elevation of EPCs in spleen. Cells from splenic tissue homogenates were lysed and analyzed with flow cytometry. EPCs in spleen were defined as CD34+/KDR+ cells (B). Co-treatment of either Phentolamine or I127 with NE significantly attenuated the increases of EPCs in spleen, but Metoprolol didn't (B, *P<0.05 compared with model group.). Representative flow cytometric analyses of EPCs (CD34+/Flk-1+cells) were showed in part B.

Male C57BL/6J mice were randomized in six groups (n = 6). Limb ischemia model was prepared in every group except control group as describe before. NE was intraperitoneally injected at 1-5 days in NE treatment group. Besides, Phentolamine (100 µl×100 µM), Metoprolol (100 µl×100 µM) and I127 (100 µl×100 µM) were also intraperitoneally injected with NE every day respectively in Phentolamine co-treatment group, Metoprolol co-treatment group and I127 co-treatment group respectively. Flow cytometric analysis showed that co-treatment of either Phentolamine or I127 with NE significantly attenuated the increases of EPCs in peripheral circulation, BM and spleen, but Metoprolol didn't(Figure 9, 10, 11). These results confirmed that NE increased EPCs mobilization via α adrenoceptor and β 2 adrenoceptor in vivo.

### Norepinephrine increased phosphorylation of Akt and eNOS of EPCs

**Figure 12:**
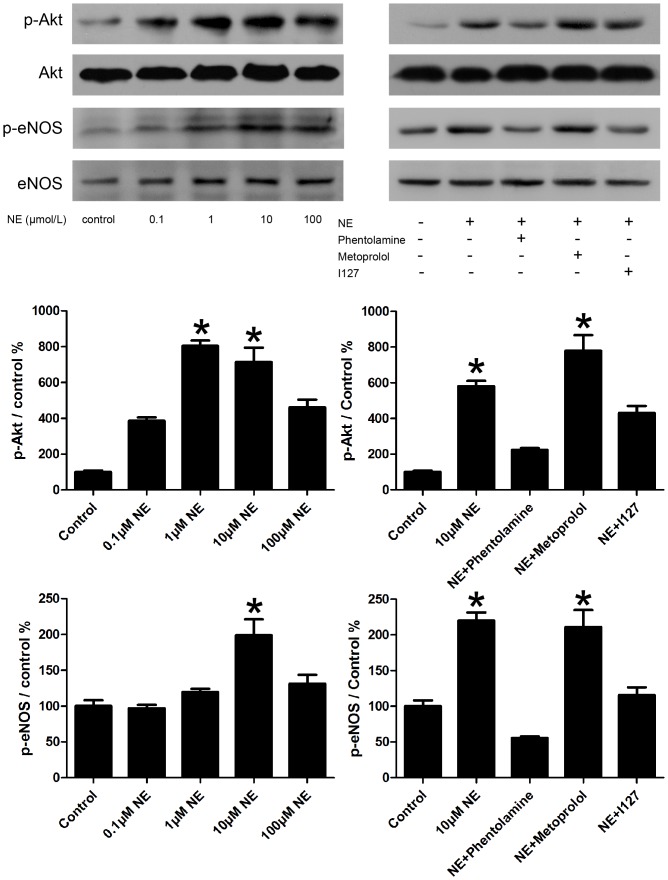
NE activated EPCs via Akt/eNOS pathways. Treatment of NE significantly increased phosphorylation of Akt, eNOS. Addition of 10 µM Phentolamine and 10 µM I127 attenuated the activation of Akt/eNOS pathway. However, addition of 10 µM Metorolol could not. Representative immunoblots (top) and densitometric quantification (bottom) demonstrated phosphorylation of Akt and eNOS in the following groups: control, 0.1 µM NE, 1 µM NE, 10 µM NE, 100 µM NE, NE plus Phentolamine, NE plus Metorolol and NE plus I127. *P<0.05 vs control.

In prior researches, the elevations of EPCs migrative activity and proliferation potential were partly regulated via the PI3-K/Akt/eNOS signaling pathway. We tested whether the treatment of NE for 24 hours could impact Akt, eNOS signal in EPCs. As showed in figure 12, treatment with NE significantly increased phosphorylation of Akt, eNOS. Addition of 10 µM Phentolamine and 10 µM I127 attenuated the activation of Akt/eNOS pathway. However, addition of 10 µM Metorolol could not.

## Discussion

Neovascularization at the ischemic tissue requires not solely angiogenesis but also circulating EPCs during vasculogenesis. An inadequate angiogenic response to ischemia in the peripheral ischemic limbs or myocardium of patients might result in poor collateral formation and severe organ damage. EPCs are currently thought to be the primary cells resource for postnatal vasculogenesis. They can be detected from several tissues, such as peripheral blood, cord blood, spleen, vessel walls and even the heart and skeletal muscle. [12] In this study, we evaluated the number of EPCs in peripheral blood, BM and spleen in mouse bearing hind limb ischemia. Results showed that NE increased EPCs number in all BM, peripheral circulation and spleen. BM which is considered to be the main hematopoietic organ and the typical source of EPCs is histologically supplied by autonomic nerve fibres[13]. The nerve cells and other cells release catecholamines in the BM. It has been reported that limb ischemia increase tyrosine hydroxylase mRNA levels and catecholamines synthesis within BM. [14] In the present study, we found that co-treatment of α adrenoceptor blocker and β 2 adrenoceptor blocker attenuated the elevation of EPCs. These results were consistent with previous studies in which in vivo adenoviral-mediated gene transfer of the human β2 adrenergic receptor improved hindlimb perfusion and capillary density.[15] Stimulation of endogenous and overexpressed β2 adrenergic receptor in vitro was also found to increase the proliferation of endothelial cells. Similar results were also reported previously by Galasso et al, who stated that β2-adrenergic receptor stimulation improves endothelial progenitor cell-mediated ischemic neoangiogenesis. [16]

The effect of NE on EPCs might be mediated by Akt/eNOS signaling pathway. The PI3K/Akt/eNOS pathway has been implicated in the mobilization of EPCs from the BM, promoting EPCs differentiation and inhibiting EPCs apoptosis. This signal pathway is impaired in many pathological conditions such as diabetes[17], hypertension[18], etc. In this study, we observed NE increased Akt/eNOS pathway in EPCs through α adrenoceptor and β 2 adrenoceptor. Akt possesses a protein domain that binds phosphoinositides, which are themselves phosphorylated by members of the phosphoinositide 3 kinase (PI3K) family. PI3-kinases may be activated by G protein-coupled receptors as adrenoceptors. Activation of Akt also appears to promote phosphorylation of eNOS, increase endothelial NO production and hence cell growth and migration. It is reported before that eNOS knockout mice that have undergone BM transplantation have a persistently reduced level of EPCs after experimentally induced hind limb ischaemia[19].

VEGF also participate in the regulation of EPCs proliferation and migration in limb ischemic mice. Our study showed that NE treatment increased VEGF concentrations in both bone marrow and skeletal muscle which might be the primary factor mediating the effect of NE on EPCs mobilization in BM microenvironment. Co-treatment of Phentolamine and I127 attenuated the increase of VEGF in bone marrow and skeletal muscle. Catecholamines were previously reported to augment capillary formation by promoting release of VEGF by ischemic skeletal muscle cells[20]. Catecholamines stimulate angiogenesis in rat brown adipose tissue by an increase in VEGF release from adipocytes[21]. Also, stimulation of adrenoceptors on cultured neonatal rat cardiac nonmyocytes releases TGF-beta and atrial natriuretic peptide into the conditioned medium, which, when presented to cardiomyocytes, upregulates VEGF expression[22]. So VEGF might play an important role in the NE induced EPCs mobilization and ischemic muscle angiogenesis.

The increases of EPCs in spleen might be attributed to redistribution of EPCs between organs after their mobilization. When EPCs are mobilized, they quickly leave the bloodstream (within 1–3 h) and lodge randomly in several tissues following a hemodynamic flow. It is believed that the spleen, lung, liver and kidneys, which have a larger blood supply, are primary tissues for circulating cell settlement. However, as many adhesion molecules and chemoattractant factors are highly expressed on the spleen endothelium, most EPCs migrate to the blood vessels in spleen called sinusoids. The redistribution of EPCs could also be observed in many other experiments in which EPCs were intravenously administered to promote tissue angiogenesis. In these experiments, EPCs transiently distributed randomly in several tissues (liver, lung and kidney), but mostly transmigrating into BM and spleen in contact with the endothelial cells surrounding the sinusoids[23]. So the spleen works as a center of the reticulo-endothelial system functioning in the storage and rapid deployment of EPCs. In present study, NE did not only increased EPCs proliferation and their number in BM, but also increased their migration capacity, so EPCs might quickly leave BM, redistributed in all tissues and lastly accumulated in spleen. These results were consistent with what was reported by Harald Engler who found that catecholamines increased myelopoiesis in the bone marrow that was paralleled by an accumulation of neutrophils and monocytes in circulation and spleen[24].

In previous studies, catecholamines were involved in angiogenesis and collateral growth in hindlimb insufficiency[25]. Perivascular administration of NE in the carotid artery wall increased neointimal growth after balloon injury in vivo[26]. The vascular growth in ischemic tissue was sharply attenuated in mice made deficient in catecholamines by gene deletion of dopamine β-hydroxylase [25]. Moreover, local and systemic blockade of adrenoceptor inhibited growth of the carotid artery, demonstrating a contribution of endogenous catecholamines to vascular wall growth after injury[26]. In present study, NE significantly increased angiogenesis in the gastrocnemius of the ligated leg. Co-treatment of α adrenoceptor blocker and β 2 adrenoceptor blocker inhibited the angiogenic processes in gastrocnemius after hindlimb ischemia. As EPCs contribute to approximately 90% of vascularisation in adult angiogenesis-defective mice[27], it is convenient to speculate that the effects of NE on gastrocnemius angiogenesis might primarily attribute to the pro-angiogenic effects of mobilized EPCs, including paracrine of pro-angiogenic factors and regeneration of endothelial cells. It is supported by previous studies which showed that catecholamines increased monocytes, including EPCs and stromal cells, accumulation in the gastrocnemius and the surrounding preexisting collaterals in the gracilis muscle. [25] However, further studies are needed to confirm the connection between EPCs and angiogenesis after NE treatment in limb ischemic muscle. Moreover, the growth of VSMCs, adventitial fibroblasts and expression of hypoxia-inducible factor-1might also contribute to catecholamines induced ischemic angiogenesis. [28]

In diabetes, it is shown that EPCs were trapped within the diabetic BM as a result of a lack of inherent sympathetic denervation that can alter circadian and release of EPCs from the BM capacity[29]. Our new findings would indicate that NE or adrenoceptor agonists could restore physiological EPC mobilization from the BM and improve vascular repair in pathological tissues.

Mental stress is commonly observed in patients with advanced cancers. Several studies have indicated that chronic stress would increase tumor growth and metastasis by promoting tumor angiogenesis[30], [31]. EPCs are able to facilitate tumor-induced vasculogenesis. It has been reported that EPCs contribute about 90% to vascularization in lymphomas grown in angiogenesis-defective Id-mutant mice in which implanted tumors rapidly regress in association with poor development of tumor neovessels[32].In this study, we observed that sympathetic neurotransmitter NE increased the mobilization of EPCs through α adrenoceptor and β 2 adrenoceptor. These might be a complement mechanism through which chronic mental stress correlates to tumor growth, metastasis and poor clinical outcomes. In another hand, the modification of adrenoceptor/Akt/eNOS pathway could be a meaningful complement for the treatment of malignant tumor.

## Conclusion

It has been shown here that the sympathetic nervous system regulates, through Akt/eNOS signaling, the mobilization of EPCs. Our study also suggests that regulation of sympathetic nervous system may constitute an innovative strategy to modulate EPCs mobilization and vessel growth in cancer or cardiovascular ischemic diseases. These findings broadened the understanding of the function of sympathetic nervous system in the regulation of the retention, proliferation and mobilization of EPCs and may inform a new mechanism that regulate progenitor cell recruitment in disease and be exploited to provide efficacious stem cell therapy for tissue regeneration.

## References

[1] Ramcharan SK, Lip GY, Stonelake PS, Blann AD (2013) Angiogenin outperforms VEGF, EPCs and CECs in predicting Dukes' and AJCC stage in colorectal cancer. European Journal of Clinical Investigation 43: 801–8. 23683169 10.1111/eci.12108

[2] Fox A, Smythe J, Fisher N, Tyler MP, McGrouther DA, et al (2008) Mobilization of endothelial progenitor cells into the circulation in burned patients. British Journal of Surgery 95: 244–51. 17702088 10.1002/bjs.5913

[3] Tanaka R, Vaynrub M, Masuda H, Ito R, Kobori M, et al (2013) Quality-control culture system restores diabetic endothelial progenitor cell vasculogenesis and accelerates wound closure. Diabetes 62: 3207–17. 23670975 10.2337/db12-1621 PMC3749357

[4] Lee SH, Lee JH, Yoo SY, Hur J, Kim HS, et al (2013) Hypoxia Inhibits Cellular Senescence to Restore the Therapeutic Potential of Old Human Endothelial Progenitor Cells via the Hypoxia-Inducible Factor-1α-TWIST-p21 Axis. Arteriosclerosis Thrombosis and Vascular Biology 33: 2407–14. 10.1161/ATVBAHA.113.301931 23928864

[5] Turgeon J, Dussault S, Maingrette F, Groleau J, Haddad P, et al (2013) Fish oil-enriched diet protects against ischemia by improving angiogenesis, endothelial progenitor cell function and postnatal neovascularization. Atherosclerosis 229: 295–303. 23880179 10.1016/j.atherosclerosis.2013.05.020

[6] Sun CK, Leu S, Sheu JJ, Tsai TH, Sung HC, et al (2013) Paradoxical impairment of angiogenesis, endothelial function and circulating number of endothelial progenitor cells in DPP4-deficient rat after critical limb ischemia. Stem Cell Research & Therapy 4: 31. 23517567 10.1186/scrt181 PMC3706813

[7] Lee JH, Lee SH, Yoo SY, Asahara T, Kwon SM (2013) CD34 hybrid cells promote endothelial colony-forming cell bioactivity and therapeutic potential for ischemic diseases. Arteriosclerosis Thrombosis and Vascular Biology 33: 1622–34. 10.1161/ATVBAHA.112.301052 23640491

[8] Galasso G, De Rosa R, Ciccarelli M, Sorriento D, Del Giudice C, et al (2013) β2-Adrenergic receptor stimulation improves endothelial progenitor cell-mediated ischemic neoangiogenesis. Circulation Research. 29 112(7): 1026–34. 10.1161/CIRCRESAHA.111.300152 23418295

[9] Récalde A, Richart A, Guérin C, Cochain C, Zouggari Y, et al (2012) Sympathetic nervous system regulates bone marrow-derived cell egress through endothelial nitric oxide synthase activation: role in postischemic tissue remodeling. Arteriosclerosis Thrombosis and Vascular Biology 32: 643–53. 10.1161/ATVBAHA.111.244392 22267478

[10] Sun C, Liang C, Ren Y, Zhen Y, He Z, et al (2009) Advanced glycation end products depress function of endothelial progenitor cells via p38 and ERK 1/2 mitogen-activated protein kinase pathways. Basic Research in Cardiology 104: 42–9. 18622638 10.1007/s00395-008-0738-8

[11] Liang C, Ren Y, Tan H, He Z, Jiang Q, et al (2009) Rosiglitazone via upregulation of Akt/eNOS pathways attenuates dysfunction of endothelial progenitor cells, induced by advanced glycation end products. British Journal of Pharmacology 158: 1865–73. 19917066 10.1111/j.1476-5381.2009.00450.x PMC2807648

[12] Iwaguro H, Yamaguchi J, Kalka C, Murasawa S, Masuda H, et al (2002) Endothelial progenitor cell vascular endothelial growth factor gene transfer for vascular regeneration. Circulation 105: 732–8. 11839630 10.1161/hc0602.103673

[13] Tabarowski Z, Gibson-Berry K, Felten S (1996) Noradrenergic and peptidergic innervation of the mouse femur bone marrow. Acta Histochem 98: 453–7. 8960309 10.1016/S0065-1281(96)80013-4

[14] Récalde A, Richart A, Guérin C, Cochain C, Zouggari Y, et al (2012) Sympathetic nervous system regulates bone marrow-derived cell egress through endothelial nitric oxide synthase activation: role in postischemic tissue remodeling. Arteriosclerosis Thrombosis and Vascular Biology 32: 643–53. 10.1161/ATVBAHA.111.244392 22267478

[15] Iaccarino G, Ciccarelli M, Sorriento D, Galasso G, Campanile A, et al (2005) Ischemic neoangiogenesis enhanced by beta2-adrenergic receptor overexpression: a novel role for the endothelial adrenergic system. Circulation Research. 25 97: 1182–9. 10.1161/01.RES.0000191541.06788.bb 16239589

[16] Galasso G, De Rosa R, Ciccarelli M, Sorriento D, Del Giudice C, et al (2013) β2-Adrenergic receptor stimulation improves endothelial progenitor cell-mediated ischemic neoangiogenesis. Circ Res 112: 1026–34. 23418295 10.1161/CIRCRESAHA.111.300152

[17] Bernal-Mizrachi E, Fatrai S, Johnson JD, Ohsugi M, Otani K, et al (2004) Defective insulin secretion and increased susceptibility to experimental diabetes are induced by reduced Akt activity in pancreatic islet beta cells. Journal of Clinical Investigation 114: 928–36. 15467831 10.1172/JCI20016 PMC518659

[18] Peng X, Haldar S, Deshpande S, Irani K, Kass DA (2003) Wall stiffness suppresses Akt/eNOS and cytoprotection in pulse-perfused endothelium. Hypertension 41: 378–81. 12574111 10.1161/01.hyp.0000049624.99844.3d

[19] Aicher A, Heeschen C, Mildner-Rihm C, Urbich C, Ihling C, et al (2003) Essential role of endothelial nitric oxide synthase for mobilization of stem and progenitor cells. Nature Medcine 9: 1370–6. 10.1038/nm948 14556003

[20] Catoire M, Mensink M, Boekschoten MV, Hangelbroek R, Müller M, et al (2012) Pronounced effects of acute endurance exercise on gene expression in resting and exercising human skeletal muscle. PLoS One 7: e51066. 23226462 10.1371/journal.pone.0051066 PMC3511348

[21] Park SY, Kang JH, Jeong KJ, Lee J, Han JW, et al (2011) Norepinephrine induces VEGF expression and angiogenesis by a hypoxia-inducible factor-1α protein-dependentmechanism. Int J Cancer 128: 2306–16. 20715173 10.1002/ijc.25589

[22] Rosenkranz S. TGF-beta1 and angiotensin networking in cardiac remodeling (2004) Cardiovasc Res. 63: 423–32. 10.1016/j.cardiores.2004.04.030 15276467

[23] Aicher A, Brenner W, Zuhayra M, Badorff C, Massoudi S, et al (2003) Assessment of the tissue distribution of transplanted human endothelial progenitor cells by radioactive labeling. Circulation 107: 2134–9. 12695305 10.1161/01.CIR.0000062649.63838.C9

[24] Engler H, Bailey MT, Engler A, Sheridan JF (2004) Effects of repeated social stress on leukocyte distribution in bone marrow, peripheral blood and spleen. J Neuroimmunol 148: 106–15. 14975591 10.1016/j.jneuroim.2003.11.011

[25] Chalothorn D, Zhang H, Clayton JA, Thomas SA, Faber JE (2005) Catecholamines augment collateral vessel growth and angiogenesis in hindlimb ischemia. Am J Physiol Heart Circ Physiol 289: H947–59. 15833801 10.1152/ajpheart.00952.2004

[26] Erami C, Zhang H, Tanoue A, Tsujimoto G, Thomas SA, et al (2005) Adrenergic catecholamine trophic activity contributes to flow-mediated arterial remodeling. Am J Physiol Heart Circ Physiol 289: H744–53. 15849236 10.1152/ajpheart.00129.2005

[27] Lyden D, Hattori K, Dias S, Costa C, Blaikie P, et al (2001) Impaired recruitment of bone-marrow-derived endothelial and hematopoietic precursor cells blocks tumour angiogenesis and growth. Nat Med 7: 1194–201. 11689883 10.1038/nm1101-1194

[28] Zhang H, Cotecchia S, Thomas SA, Tanoue A, Tsujimoto G, et al (2004) Gene deletion of dopamine β-hydroxylase and α1-adrenoceptors demonstrates involvement of catecholamines in vascular remodeling. Am J Physiol Heart Circ Physiol 287: H2106–14. 15231500 10.1152/ajpheart.00290.2004

[29] Busik JV, Tikhonenko M, Bhatwadekar A, Opreanu M, Yakubova N, et al (2009) Diabetic retinopathy is associated with bone marrow neuropathy and a depressed peripheralclock. Journal of Experimental Medicine. 206: 2897–906. 10.1084/jem.20090889 PMC2806461 19934019

[30] Thaker PH, Han LY, Kamat AA, Arevalo JM, Takahashi R, et al (2006) Chronic stress promotes tumor growth and angiogenesis in a mouse model of ovarian carcinoma. Nature Medcine 12: 939–44. 10.1038/nm1447 16862152

[31] Qijun J, Zhigang G, Shifang D (2013) Endothelial progenitor cells may participate in stress-induced tumour angiogenesis. Medical Hypotheses 80: 778–780. 23578361 10.1016/j.mehy.2013.03.010

[32] Lyden D, Hattori K, Dias S, Costa C, Blaikie P, et al (2001) Impaired recruitment of bone-marrow-derived endothelial and hematopoietic precursor cells blocks tumor angiogenesis and growth. Nature Medcine 7: 1194–201. 10.1038/nm1101-1194 11689883

